# Pan-European meteorological and snow indicators of climate change impact on ski tourism

**DOI:** 10.1016/j.cliser.2021.100215

**Published:** 2021-04

**Authors:** Samuel Morin, Raphaëlle Samacoïts, Hugues François, Carlo M. Carmagnola, Bruno Abegg, O. Cenk Demiroglu, Marc Pons, Jean-Michel Soubeyroux, Matthieu Lafaysse, Sam Franklin, Guy Griffiths, Debbie Kite, Anna Amacher Hoppler, Emmanuelle George, Carlo Buontempo, Samuel Almond, Ghislain Dubois, Adeline Cauchy

**Affiliations:** a Univ.Grenoble Alpes, Université de Toulouse, Météo-France, CNRS, CNRM, CEN, 38000 Grenoble, France; b Météo-France, Direction de la Climatologie et des Services Climatiques, Toulouse, France; c Univ.Grenoble Alpes, INRAE, LESSEM, Grenoble, France; d University of St. Gallen, Institute for Systemic Management and Public Governance, St. Gallen, Switzerland; e Umeå University, Department of Geography, Umeå, Sweden; f Snow and Mountain Research Center of Andorra/ Sustainability Observatory of Andorra, Sant Julià, Andorra; g The Institute for Environmental Analytics (IEA), University of Reading, Reading, United Kingdom; h Lucerne University of Applied Sciences and Arts, Institute of Tourism, Lucerne, Switzerland; i European Center for Medium-range Weather Forecasts, Reading, United Kingdom; j TEC Conseil – Ramboll, Aix en Provence, France

**Keywords:** Ski tourism, Mountain tourism, Climate change, Europe, Copernicus

## Abstract

Ski tourism plays a major socio-economic role in the snowy and mountainous areas of Europe such as the Alps, the Pyrenees, Nordic Europe, Eastern Europe, Anatolia, etc. Past and future climate change has an impact on the operating conditions of ski resorts, due to their reliance on natural snowfall and favorable conditions for snowmaking. However, there is currently a lack of assessment of past and future operating conditions of ski resorts at the pan-European scale in the context of climate change. The presented work aims at filling this gap, as part of the ”European Tourism” Sectoral Information System (SIS) of the Copernicus Climate Change Services (C3S). The Mountain Tourism Meteorological and Snow Indicators (MTMSI) were co-designed with representatives of the ski tourism industry, including consulting companies. They were derived from statistically adjusted EURO-CORDEX climate projections (multiple GCM/RCM pairs for RCP2.6, RCP4.5 and RCP8.5) using the UERRA 5.5 km resolution surface reanalysis as a reference, used as input to the snow cover model Crocus, with and without accounting for snow management (grooming, snowmaking). Results are generated for 100 m elevation bands for NUTS-3 geographical areas spanning all areas relevant to ski tourism in Europe. This article introduces the underpinning elements for the generation of this product, and illustrates results at the pan-European scale as well as for smaller scale case studies. A dedicated visualization app allows for easy navigation into the multiple dimensions of this dataset, thereby fulfilling the needs of a broad range of users.

## Highlights

• Provision of pan-European climate change impact indicators for ski tourism.
• The indicators account for natural snow cover processes, grooming and snowmaking.
• The indicators are provided for NUTS-3 areas by elevation steps of 100 m.
• The indicators combine reanalysis and regional climate model projections (1950–2100).
• The indicators are available freely through the Copernicus C3S Climate Data Store.

**Table:** 

Practical implications
Ski tourism plays a major socio-economic role in the snowy and mountainous areas of Europe such as the Alps, the Pyrenees, Nordic Europe, Eastern Europe, Anatolia, etc. Meteorological conditions govern the operating conditions of ski resorts, due to their reliance on natural snowfall and favourable conditions for snowmaking. However, there is currently a lack of assessment of past and future operating conditions of ski resorts at the pan-European scale in the context of climate change in a homogeneous way, which would allow a pan-European appraisal of changes and impacts and enable relevant comparisons across European regions. Multiple studies have evidenced for several decades, at local and national scales, that ski tourism is under a major climate threat, because of increasing air temperature and natural snow scarcity at low elevation. However, many studies, especially in earlier times or with a regional scope, did not account for snowmaking in their calculations, leading to results not directly appropriate for addressing the impact of climate change on the ski tourism industry because they did not reflect growing and nowadays routine operational practices of the industry. This shortcoming has been identified in recent scientific studies and accounted for in recent policy-relevant assessments (e.g. IPCC Special Report on 1.5°C Global Warming, 2018, and Special Report on the Ocean and Cryosphere in a Changing Climate, 2019). Nevertheless, this has led to some confusion at the local level, because the simulated impact of climate change on ski tourism depends very much on whether and how snow management is accounted for in the modelling chains, and methodological choices (e.g. regarding regional climate modelling with or without additional downscaling, climate change scenarios etc.) (Abegg et al., 2020).
The presented work aims at filling this gap, as part of the “European Tourism” Sectoral Information System (SIS) of the Copernicus Climate Change Services (C3S). C3S is run by the European Center for Medium-Range Weather Forecast (ECMWF) on behalf of the European Commission. The Mountain Tourism Meteorological and Snow Indicators (MTMSI) were defined based on scientific literature and refined through structured interviews with stakeholders from the ski tourism industry, thereby enabling co-design with relevant users. They were computed from statistically adjusted EURO-CORDEX climate projections (9 GCM/RCM pairs for Representative Concentration Pathways RCP4.5 and RCP8.5 and 2 for RCP2.6) using the UERRA 5.5 km resolution surface reanalysis as a reference, followed by model runs of the snow cover model Crocus, with and without accounting for snow management (grooming, snowmaking). Results were produced based on reanalysis from 1960 to 2015, and for climate change projections using the historical time period from 1950 to 2005 and for future scenarios from 2006 to 2100. Results were generated for 100 m elevation bands for NUTS-3 (2016) geographical areas spanning all areas relevant for ski tourism in Europe. A series of 39 indicators were computed, with one value per year (e.g., (i) number of days with snow depth above a given threshold, for various configurations, i.e. natural snow cover, groomed snow cover, or managed snow cover, combining the effects of natural processes, grooming, and snowmaking, for a given time period during the year, or (ii) mean air temperature during a given time period), representing synthetic indicators making it possible to address various questions related to climate change and the ski tourism industry. The annual-scale data can be aggregated in time and space in various ways, depending on the use case.
This article introduces the underpinning elements for the generation of this product, and illustrates results at the pan-European scale as well as three case studies at smaller scales, for past and future climate conditions. For example, the data were used by the consultancy company ATC to assess the impact of climate change on snow reliability and contribute to shaping infrastructure and diversification choices for a ski tourism destination in Western Turkey. In Andorra, this data set was used in collaboration with the National Energy and Climate Change Office of the Andorra Government, to explore how it could contribute to the National Climate Change Adaptation Strategy, specifially to assess and design the necessity of sectoral adaptation strategies. The dataset was also tested by the Swiss association of cable car companies (SBS).
The dataset and the visualization App have been be made available freely through the Copernicus Data Store in 2020. The C3S European Tourism MTMSI is not meant to replace higher resolution products which are available in some European countries, and provide a more detailed view of the future of snow conditions in European ski resorts, accounting, for example, for slope, aspect, local phenomena and local snow management practices. However, given that the workflow for the generation of the product is homogeneous at the pan-European level, it makes it a useful tool to assess the main features of past and future snow conditions at the pan-European level, or to compare distant destinations (e.g., compare Scandinavia and Eastern Europe for a given elevation and time horizon). Furthermore, where no other source of information is currently available, it provides an original outlook on future meteorological and snow conditions in mountain areas.
It is expected that the data and the tools developed within this project will not only make it possible to analyze the climate sensitivity of ski tourism in Europe as a topical yet academic research question, but will also help third-parties in developing climate services specifically targeting the ski tourism industry in Europe.

## 1 Introduction

Like most human activities, tourism depends on meteorological conditions, and is hence impacted by climate change. Ski tourism is one of the most often cited example for such a link between climate change and tourism, because climate projections indicate a significant reduction of seasonal snow in low elevation mountain areas (Hock et al., in press, and references therein). Ski tourism plays a major role in the socio-economic functioning of European mountain areas such as the Alps (leading ski tourism destination worldwide, Vanat, 2020), Pyrenees, Scandinavia, Anatolia, Eastern European Mountains etc. Meteorological conditions govern the operating conditions of ski resorts, due to their reliance on natural snowfall and favorable conditions for snowmaking. They are thus sensitive to interannual variability of snow conditions, and long term climate change, addressed mostly in a number of studies at the local or national scale (Steiger et al., 2019, and references therein). There is currently a major lack of assessment of past and future operating conditions of ski resorts at the pan-European scale in the context of climate change. Most pan-European (Beniston et al., 2018) or regional (e.g. Gobiet et al., 2014) climate change studies address past and future change of meteorological conditions and natural snow conditions. Such information provides context for ski resorts operating conditions, and have been used to infer future ski tourism projections in many European countries (Damm et al., 2017, Tranos and Davoudi, 2014). Still, their relevance for ski tourism stakeholders is limited, in particular because such studies do not account for snow management, although it plays a central role for the operations of ski resorts (Steiger et al., 2019, Hock et al., in press, Hanzer et al., 2020). In contrast, several studies have provided information relevant to ski resorts management at the local to regional scale, accounting for future climate conditions but also explicitly handling snow management elements, such as threshold wet-bulb temperature for snow making, impact of grooming etc. (Marke et al., 2014, Pons et al., 2015, Spandre et al., 2019a, Spandre et al., 2019b, Scott et al., 2019, Steiger and Scott, 2020). Such studies, however, are based on different tools and hypotheses, not always in line with state-of-the-art practices for climate change impact studies, which makes it difficult to compare them and compile their results into a pan-European outlook to climate change impacts on ski tourism (Abegg et al., 2020). Table 1 summarizes the methods employed in several recent studies addressing future climate change impacts to ski tourism in Europe, making it possible to highlight a few commonalities and many differences between these studies. The “Mountain” component of the “European Tourism” Sectoral Information Service (SIS) of Copernicus Climate Change Services (C3S) intends to address this knowledge gap. This contribution introduces the scientific background and approach for generating the C3S SIS European Tourism ”Mountain component” products, and delivers the main results relevant to this operational service, opened to users since 2020.

**Table 1:** Synthesis of approaches used for recent regional or local scale climate change impact studies on ski tourism in Europe.

Reference	Geographical domain	Geographical resolution	Representation of ski resorts	Impact model	Indicators	Downscaling method	Climate projections	Statistical processing
Damm et al. (2017)	12 European countries (AT, CH, CZ, DE, ES, FI, FR, IT, SE, SI, SK, NO)	NUTS-3 (3 simulation points per NUTS-3 at low, mean and high elevation, on flat terrain)	Location of ski resorts accounted for in the calculation of NUTS-3 representative elevations.	Hydrological model VIC (natural snow processes)	Monthly mean snow water equivalents, Fraction of days per month with at least 120 mm SWE, Fraction of days per month with at least 4 mm SWE	Use of E-OBS data for current conditions. No downscaling for future changes.	11 EURO-CORDEX pairs (2x RCP2.6, 5x RCP4.5, 4x RCP8.5)	Median
Tranos and Davoudi (2014)	27 EU countries + Switzerland, Norway, Iceland and Liechtenstein	NUTS-3, one climate model grid point per NUTS-3	No representation of ski resorts	Natural snow model in CCLM climate model	Number of days with snow cover	Direct use of regional climate output	1 CCLM model run (A1B, driving GCM not stated).	Unknown.
Spandre et al. (2019a)	FR (French Alps)	Massifs (1000 km2), with elevation steps of 300 m, accounting for several slopes and aspects.	Explicit representation of ski resorts’ topography and spatial organization in the model chain.	Crocus-Resort, accounting for natural, grooming and snowmaking processes	Resort-level reliability computed over Christmas and Winter seasons, based on the proportion of the ski resorts with at least 100 kg m-2 SWE.	ADAMONT method, use of SAFRAN reanalysis as observation data set.	30 EURO-CORDEX pairs (4x RCP2.6, 13x RCP4.5, 13x RCP8.5)	Multi-model mean/stdev and quantiles of annual values for selected time periods.
Spandre et al. (2019b)	FR, ES, AD (French Alps and Pyrenees),	Massifs (1000 km2), with elevation steps of 300 m, on flat terrain.	Explicit representation of location and elevation range of ski resorts for the computation of the reliability categories.	Crocus-Resort, accounting for natural, grooming and snowmaking processes	Reliability elevation line based on number of days with at least 100 kg m^−2^ SWE (with and without snowmaking)	ADAMONT method, use of SAFRAN reanalysis as observation data set.	30 EURO-CORDEX pairs (4x RCP2.6, 13x RCP4.5, 13x RCP8.5)	Classification of ski resorts based on reliability categories, based on distribution of annual values, depending on time periods.
Steiger and Scott (2020)	AT	Simulations for 208 ski resorts in Austria, results reported for 7 provinces and full country.	Explicit simulation for each ski area, using 100 m elevation bands and three aspects (north, south, west/east), and adjusting the snowmaking intensity based on comparison with observed opening/closure dates.	SkiSim3, calibrated using observations in ski resorts.	Snow depth above 30 cm for at least 100 days or snow depth continuously above 30 cm during the Christmas-New Years holiday period in 7 out of 10 seasons. Terrain indicator based on snow conditions at different time periods during the snow season.	56 weather stations (ZAMG) for baseline simulations; use of monthly temperature and precipitation changes from climate projections.	26 EURO-CORDEX pairs (13x RCP4.5, 13xRCP8.5)	Ensemble means for various 30 years time periods in the future
Scott et al. (2019)	NO	Simulations for 110 ski resorts in Norway, results reported for 5 provinces.	Explicit simulation for each ski area, using 100 m elevation bands and three aspects (north, south, west/east), and adjusting the snowmaking intensity based on comparison with observed opening/closure dates.	SkiSim2	Snow depth above 30 cm for at least 100 days	41 weather stations for baseline simulations; change values from climate projections.	20 EURO-CORDEX pairs (10x RCP4.5, 10xRCP8.5)	Ensemble average values for various 30 years time periods in the future

Section 2 introduces the indicators and the geographical, temporal and technical configuration for their computation, based on the structure of Table 1. Section 3 introduces the outcome of the computation, including the uses of the indicators in several case studies. Section 4 discusses the main limitations and considerations to be accounted for in the use of the indicators. Section 5 concludes and outlines future activities and studies that the indicators allow.

## 2 Material and methods

This section introduces the design and features of the MTMSI data set, following the same structure as Table 1, i.e. Geographical domain (2.2), Geographical resolution (2.3), Representation of ski resorts (2.4), Impact model (2.5), Indicators (2.6), Downscaling method (2.7), Climate projections (2.8) and Statistical post-processing (2.9).

### 2.1 Co-design approach

A previous C3S contract (SECTEUR, 2016–2017) already pre-identified key requirements and stakeholders for the tourism sector, especially for the ski tourism industry, through extensive user consultation (European survey, workshops) across multiples sectors. Among core missing indicators across Europe were the snow reliability indicators for past, current and future climate including natural and artificial snow conditions. Intermediaries, such as consultancy companies operating in the mountains of Europe, were considered as potential key stakeholders. Building upon the SECTEUR project, C3S European Tourism implemented a user-driven system by engaging with mountain stakeholders in the design and the implementation of the system composed of datasets, applications and case studies. Complementary to a literature review, in-depth interviews with key potential users were conducted during the scoping stage allowing an update and refinement of key requirements in respect to the snow conditions (key thresholds etc.). A total of 8 interviews were carried out, which addressed representatives from the ski industry, consultants, NGOs and weather and climate service providers. During the implementation stage, consultation mainly focused on assessing the fitness for purpose of the application being developed and on developing case studies illustrating potential uses of the service by different type of users, including intermediaries.

### 2.2 Geographical domain

The MTMSI dataset was developed to cover the largest possible fraction of the pan-European domain. It includes the European Union, candidate countries and members of the European Free Trade Association. Altogether, the dataset covers EU member states, Albania, Andorra, Montenegro, North Macedonia, Serbia, Turkey, the United Kingdom, Iceland, Liechtenstein, Norway and Switzerland.

### 2.3 Geographical resolution

**Fig. 1:**
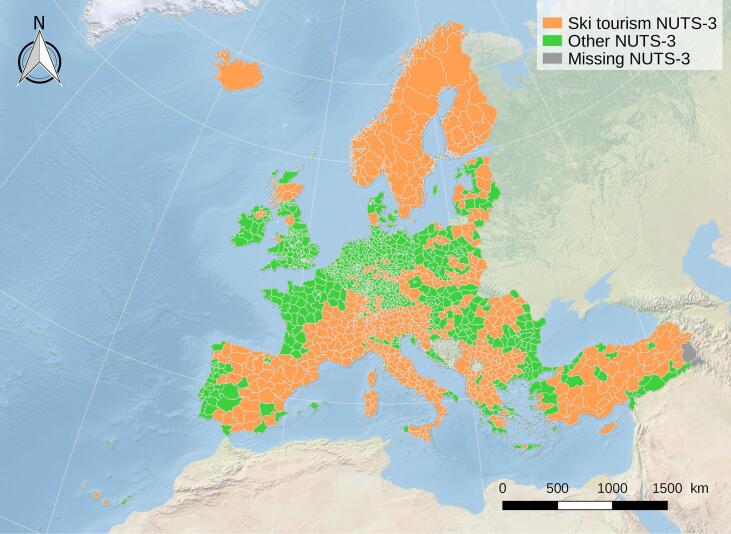
Overview of the NUTS-3 areas classified as holding ski tourism character, or not.

**Fig. 2:**
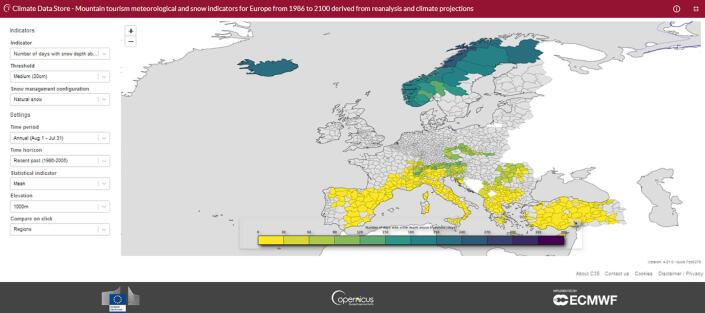
Overview of the App used to navigate through the dataset. The map displays values only for NUTS-3 areas where the selected elevation is included in the dataset.

The indicators were computed at the geographical scale of NUTS-3 areas (2016 version, see Fig. 1), in order to provide European-wide information, and the need expressed by some interviewed stakeholders to be able to link climate information with other socio-economic information, consistent with previous studies (e.g., Damm et al., 2017, Tranos and Davoudi, 2014) (see Fig. 2).

A combination of geographical information system (GIS) approaches with expert analysis of regions hosting significant ski tourism across Europe, was implemented in order to select, out of the 1519 NUTS-3 areas in Europe, those that should be considered for ski tourism purposes. Although not formally considered a NUTS-3 area, Andorra was included as a whole as a NUTS-3 area. A total of 585 NUTS-3 areas were selected for their mountainous character and ski tourism potential. Fig. 1 illustrates NUTS-3 areas of Europe according to their assigned ski tourism character, defined for the purpose of the present work.

The primary meteorological information used to compute the indicators (2.6) for past and future climate conditions 2.7, 2.8 is the UERRA 5.5 km surface reanalysis (Soci et al., 2016), which is the highest-resolution possible reanalysis data set at European scale.

Based on interviews with stakeholders (and consistent with the literature), the elevation range attracting most attention and critical challenges lies between 1000 and 2000 m elevation in alpine areas (Alps, Pyrenees), but can be significantly lower, particularly in northern regions or higher in southern regions. Indicators were required to be computed every 100 m elevation. Hence a number of 100 m elevation bands were defined for each ski-tourism NUTS-3 area, based on an analysis of their elevation range using a 30 m European-scale Digital Elevation Model, made available by the Copernicus service EUDEM v1.1 based on ASTER and SRTM images (https://land.copernicus.eu/imagery-in-situ/eu-dem/eu-dem-v1.1). For other NUTS-3 areas, the mean elevation was calculated and rounded to the closest 100 m elevation band. For each NUTS-3 area, and each 100 m elevation band considered, a mechanism was implemented to identify the data point, in the UERRA 5.5 km reanalysis topographical grid, matching the elevation band within the NUTS-3 area, with a buffer of 10 km around the boundaries of the NUTS-3 area. This method assumes that, within a NUTS-3 area, changes in climate condition arise mostly from elevation changes and to a second degree from horizontal distance, in a manner comparable to the SAFRAN system implemented from French mountainous areas (Durand et al., 2009). The implications and limitations from this choice are discussed in Section 4.

Following this geographical analysis step, surface atmospheric variables of the UERRA 5.5 km reanalysis (Soci et al., 2016) were used, for 5652 points selected depending on their location and elevation, within NUTS-3 areas. In addition, 932 points were selected to cover all European NUTS-3 areas, at their mean elevation. Note that while UERRA 5.5 km points all have different coordinates, within a given NUTS-3 area they were all assigned the same latitude and longitude (barycenter of the NUTS-3), and the elevation was rounded to the nearest 100 m elevation band.

### 2.4 Representation of ski resorts

This work does not explicitly represent ski resorts within each NUTS-3 area. Indicators provided for each NUTS-3 area and elevation bands can be combined, together with ski resorts geographical characteristics, but this is beyond the scope of this study.

### 2.5 Impact model

In order to compute indicators relevant to the snow cover, the Crocus snow cover model was used (Vionnet et al., 2012) and fed by meteorological data from past and future climate conditions. All simulations were carried out for flat terrain topographic configuration. Crocus makes it possible to account for grooming and snowmaking (Spandre et al., 2016), based on physical representation of these snow management practices and operational rules (Spandre et al., 2016, Spandre et al., 2019a, Spandre et al., 2019b). For the simulations with snowmaking, the maximum wind speed threshold was set to 4.2 m s^−1^, the density of machine-made snow was set to 600 kg m^−3^, and the production rate of machine made snow was set to 1.2 10^−3^ kg m^−2^  s^−1^, consistent with previous studies (e.g., Spandre et al., 2019a), and the wet-bulb temperature threshold for snowmaking was set to −5°C. In the simulations, between November 1 and December 15, up to 150 kg m^−2^ machine-made snow is produced, weather conditions permitting and regardless of natural snowfalls during the period, which corresponds to 25 cm snow depth. Between December 15 and February 28, snow is produced if meteorologically possible so as to maintain a total snow depth of 60 cm. After March 1, no more snow is produced. These threshold values are consistent with typical practices of ski resorts operators (see e.g. Spandre et al., 2016).

### 2.6 Indicators

Annual-scale indicators relevant to characterize the relationship between climate conditions and ski tourism operating conditions were computed, based on typical approaches used in the literature, confirmed through interviews with several ski tourism stakeholders (Abegg et al., 2020). A total of 39 annual-scale indicators were defined (i.e., one indicator value per calendar year), subdivided in 7 groups, depending on the main processes and variables upon which they are based:

• PR: precipitation (cumulative values of total and snow precipitation)
• Tas: temperature (mean monthly or seasonal values)
• SD: snow depth (number of days exceeding a snow depth threshold, at the seasonal scale and also for periods of high tourism interest such as the early December festivities (e.g. ”Purisima” in Spain), and the Christmas period; snow depth indicators are typically computed for natural snow, groomed snow and managed snow, which accounts for both grooming and snowmaking)
• SWE: snow water equivalent (same type of indicators as for snow depth, but using snow water equivalent (SWE) thresholds, referring to snow mass instead of snow depth)
• MM-PROD: amount of snow produced (at the seasonal scale)
• WBT: wet bulb temperature (number of hours below typical wet bulb temperature thresholds for snowmaking)
• BS-ES: beginning and end of season (estimation of onset and end of the continuous snow cover, computed for various snow management configurations)

The full list of indicators and their specific definition is provided in Table 2.

**Table 2:** List and definition of the 39 indicators.

Category	Name	Definition
PR	snowfall-amount-winter	Sum of snow precipitation, from November of year N to following April (included)
	precipitation-amount-winter:	Sum of total precipitation, from November of year N to following April (included)
Tas	tas-11:	Mean temperature for November of year N
	tas-12:	Mean temperature for December of year N
	tas-01:	Mean temperature for January of year N
	tas-02:	Mean temperature for February of year N
	tas-03:	Mean temperature for March of year N
	tas-04:	Mean temperature for April of year N
	tas-winter:	Mean temperature for November of year N to April of year N + 1 (included)
SD	sd-days-05-NS	Number of days with at least 5 cm of natural snow on the ground, starting on August 1st of year N to July 31st of year N + 1
	sd-days-05-GS	Number of days with at least 5 cm of groomed snow on the ground, starting on August 1st of year N to July 31st of year N + 1
	sd-days-05-MS	Number of days with at least 5 cm of managed snow on the ground, starting on August 1st of year N to July 31st of year N + 1
	sd-days-30-NS	Number of days with at least 30 cm of natural snow on the ground, starting on August 1st of year N to July 31st of year N + 1
	sd-days-30-GS	Number of days with at least 30 cm of groomed snow on the ground, starting on August 1st of year N to July 31st of year N + 1
	sd-days-30-MS	Number of days with at least 30 cm of managed snow on the ground, starting on August 1st of year N to July 31st of year N + 1
	sd-days-50-NS	Number of days with at least 50 cm of natural snow on the ground, starting on August 1st of year N to July 31st of year N + 1
	sd-days-50-GS	Number of days with at least 50 cm of groomed snow on the ground, starting on August 1st of year N to July 31st of year N + 1
	sd-days-50-MS	Number of days with at least 50 cm of managed snow on the ground, starting on August 1st of year N to July 31st of year N + 1
	sd-days-Xmas-NS	Number of days with at least 30 cm of natural snow on the ground, from December 22 of year N to January 4 (included) of year N + 1
	sd-days-Xmas-GS	Number of days with at least 30 cm of groomed snow on the ground, from December 22 of year N to January 4 (included) of year N + 1
	sd-days-Xmas-MS	Number of days with at least 30 cm of managed snow on the ground, from December 22 of year N to January 4 (included) of year N + 1
	sd-days-PUR-NS	Number of days with at least 30 cm of natural snow on the ground, from December 4 of year N to December 10 of year N (included)
	sd-days-PUR-GS	Number of days with at least 30 cm of groomed snow on the ground, from December 4 of year N to December 10 of year N (included)
	sd-days-PUR-MS	Number of days with at least 30 cm of managed snow on the ground, from December 4 of year N to December 10 of year N (included)
SWE	swe-days-100-NS	Number of days with an amount of at least 100 kg m^−2^ of natural snow on the ground, starting on August 1st of year N to July 31st of year N + 1
	swe-days-100-GS	Number of days with an amount of at least 100 kg m^−2^ of groomed snow on the ground, starting on August 1st of year N to July 31st of year N + 1
	swe-days-100-MS	Number of days with an amount of at least 100 kg m^−2^ of managed snow on the ground, starting on August 1st of year N to July 31st of year N + 1
	swe-days-120-NS	Number of days with an amount of at least 120 kg m^−2^ of natural snow on the ground, starting on August 1st of year N to July 31st of year N + 1
	swe-days-120-GS	Number of days with an amount of at least 120 kg m^−2^ of groomed snow on the ground, starting on August 1st of year N to July 31st of year N + 1
	swe-days-120-MS	Number of days with an amount of at least 120 kg m^−2^ of managed snow on the ground, starting on August 1st of year N to July 31st of year N + 1
MM-PROD	mm-prod	Annual amount of machine made snow produced (in kg m^−2^), from August 1st of year N to July 31st of year N + 1
WBT:	wbt-2-hrs	Early season potential snowmaking hours (for wet bulb temperature lower than −2°C), from November 1st, year N to December 31st, year N.
	wbt-5-hrs	Early season potential snowmaking hours (for wet bulb temperature lower than −5°C), from November 1st, year N to December 31st, year N.
BS-ES:	beginning-season-30-NS	Beginning of season, i.e. first date of the longest continuous period with at least 30 cm of natural snow on the ground (from August 1st of year N to July 31st of year N + 1)
	end-season-30-NS	End of season, i.e. last date of the longest continuous period with at least 30 cm of natural snow on the ground (from August 1st of year N to July 31st of year N + 1)
	beginning-season-30-GS	Beginning of season, i.e. first date of the longest continuous period with at least 30 cm of groomed snow on the ground (from August 1st of year N to July 31st of year N + 1)
	end-season-30-GS	End of season, i.e. last date of the longest continuous period with at least 30 cm of groomed snow on the ground (from August 1st of year N to July 31st of year N + 1)
	beginning-season-30-MS	Beginning of season, i.e. first date of the longest continuous period with at least 30 cm of managed snow on the ground (from August 1st of year N to July 31st of year N + 1)
	end-season-30-MS	End of season, i.e. last date of the longest continuous period with at least 30 cm of managed snow on the ground (from August 1st of year N to July 31st of year N + 1)

### 2.7 Downscaling method

In order to use regional climate projections and reducing inevitable biases prior to running impact models, the ADAMONT method (Verfaillie et al., 2017) was used to adjust the EURO-CORDEX GCM/RCM pairs (see Table 3 and Section 2.8) using the UERRA 5.5 km reanalysis as an observation reference (Soci et al., 2016). This method is based on quantile mapping applied to daily data. Quantile mapping functions were computed as a function of weather type (4 weather types considered, based on synoptic fields from 500 hPa geopotential height for the driving GCM) and season (4 seasons: DJF, MAM, JJA, SON). The adjustment was performed using UERRA 5.5 km data from 01/01/1980 to 01/01/2012 and GCM/RCM pairs from 01/01/1974 to 01/01/2006. In the special case of the GCM/RCM pair involving the MOHC–HadGEM2-ES GCM only, due to unavailable GCM data, required for weather type identification, before 1980, here are the time periods used for the adjustment: UERRA 5.5 km data from 01/01/1987 to 01/01/2012 and MOHC–HadGEM2-ES/RCM pairs from 01/01/1981 to 01/01/2006.

**Table 3:** Overview of EURO-CORDEX GCM/RCM pairs used and related RCP.

RCM	GCM	RCP2.6	RCP4.5	RCP8.5
SMHI-RCA4	MOHC–Hadgem2-ES		X	X
CNRM-ALADIN53	CNRM-CERFACS-CNRM-CM5		X	X
IPSL-INERIS-WRF331F	IPSL-CM5A-MR		X	X
MPI-CSC-REMO2009	MPI-M-MPI-ESM-LR	X	X	X
SMHI-RCA4	ICHEC-EC-EARTH	X	X	X
SMHI-RCA4	CNRM-CERFACS-CNRM-CM5		X	X
SMHI-RCA4	IPSL-CM5A-MR		X	X
SMHI-RCA4	MPI-M-MPI-ESM-LR		X	X
CCLM4-8–17	MPI-M-MPI-ESM-LR		X	X

As indicated above, all UERRA 5.5 km points within a given NUTS-3 were assigned the same latitude and longitude, hence they all have the same corresponding GCM/RCM point (for a given GCM/RCM grid geometry). This ensures consistency, within a given NUTS-3 area, of the climate change signal, although this may inhibit potential elevation dependent signals inherited from the GCM/RCM. This approach is similar to previous studies carried out in French mountain areas (Verfaillie et al., 2017, Verfaillie et al., 2018, Spandre et al., 2019a, Spandre et al., 2019b).

Following the application of the quantile mapping on daily data, 6-hourly data sets were generated following a disaggregation method using reanalysis data as guess data for the shape of the diurnal cycle (Verfaillie et al., 2017).

Reanalysis and adjusted climate projections were used as such for the computation of atmospheric indicators (e.g., temperature, wet bulb temperature, precipitation).

### 2.8 Climate projections

Atmospheric fields of GCM/RCM pairs from the EURO-CORDEX dataset were used, as indicated in Table 3, generally spanning the time period from 1950 to 2005 for historical simulations and 2006 to 2100 for future climate simulations using several Representative Concentration Pathways. Data from the EUR-11 ensemble (12.5 km resolution) were used. The selection of GCM/RCM pairs was based on previous work by Vautard et al. (2016) and complemented by additional available GCM/RCM pairs. Altogether, this comprises 20 future climate change scenarios for the 21st century, corresponding to 2305 model years (taking into account the reanalysis).

### 2.9 Statistical post-processing

Taking into account the multiple datasets used to generate them, a total of 91065 annual scale indicators were computed (with a value for each of the 6584 points). For each indicator, multi-annual/multi-model aggregated values were computed for various 30 and 20 years time periods, described below.

Aggregated data were computed as follows:

• Values for the period 1961–1990 and 1990–2015 based on reanalysis data.
• Values for the period 1986–2005, 2021–2040, 2041–2060 and 2081–2100 for GCM/RCM data.

For each of these 20 or 30 years periods, the following statistics were computed:

• Mean and standard deviation (across GCM/RCM pairs for a given RCP) for multi-annual averages
• Quantile of annual values (Q10, Q20, Q50, Q80 and Q90) across all available GCM/RCM pairs for a given time period and RCP.

Note that the mean and standard deviation for RCP2.6 values are only based on 2 available GCM/RCM pairs (9 pairs for RCP4.5 and RCP8.5).

### 2.10 MTMSI app

The MTMSI data were developed as part of the Sectoral Information System element of the Copernicus Climate Change Service (C3S). The aim of the SIS is to make climate data more accessible and easy to use for experts outside of the climate science community. Hence as well as developing the dataset, the project also developed a web-based application (app) to explore and visualize the data.

The functionality of the app was driven by an understanding of the questions that users are looking to answer using these data. For instance:

• How often are conditions below a certain threshold (e.g. number of snow-covered days)? Does this change in the future?
• Will there be snow at Christmas in the future? Either natural or man-made?
• How do ”my resorts” (say, all Austrian resorts) compare with others in the Alps at the same altitude?

While the data are all structured in the same way, which helps greatly with visualization and data handling, there are very many choices the user can make about each indicator. The data are visualized on a coloured map, with one value per NUTS3 area. For instance, if the user wishes to view the ”number of days with snow depth above a threshold”, that threshold can be chosen (5 cm, 30 cm or 50 cm), as well as the snow management regime - natural snow, groomed snow, or snowmaking? Additionally, there are several time periods that can be viewed - the whole year (1 Aug to 31 Jul), or just the Christmas period, or the ”Purisima” days (4–10 Dec). The users can also select whether they are interested in data from the recent past (1986–2005) or one of the future climate scenarios (out to 2100). Finally, the visualization can show the mean over that time horizon, the standard deviation, or a quantile from the distribution of all annual values in that period. These options vary depending on the original indicator selected.

Two more key parameters to explore are the elevation - all indicators are available in 100 m steps - and the location. If a user selects two of the NUTS3 areas, they are offered a ”Compare” button, which brings up a pyramid plot, showing the values of the chosen indicator at all available altitudes for the two locations.

The App has been developed online, directly linked to the MTMSI dataset and is available on the C3S Climate Data Store website: https://cds.climate.copernicus.eu/cdsapp#!/software/app-tourism-mountain-indicators-projections?tab=overview. This link also provides direct access to the source code of the application, enabling the design and implementation of new applications developed on the C3S Climate Data Store based on this dataset.

## 3 Results

### 3.1 Time series

**Fig. 3:**
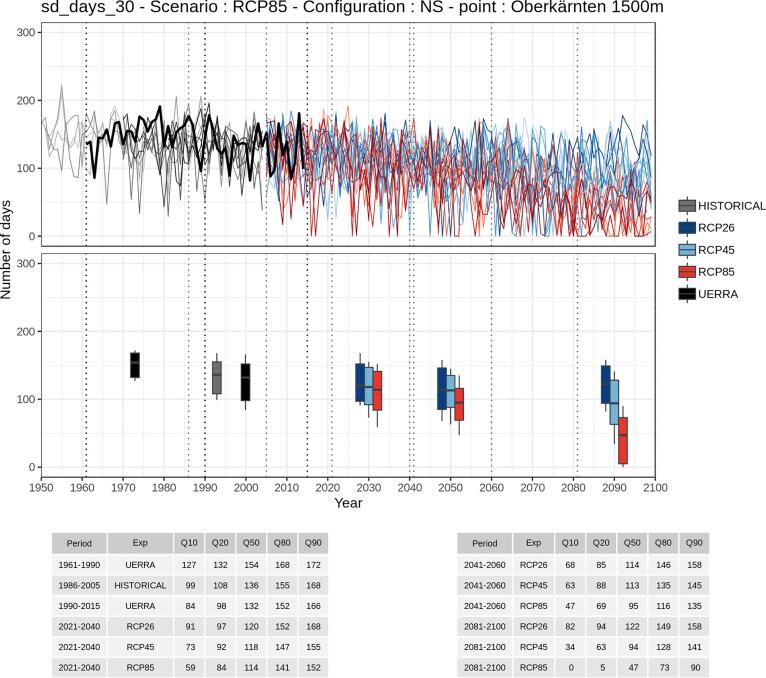
Time series of the annual number of days with more than 30 cm of natural snow at 1500 m elevation, for the Oberkärnten (Upper Carinthia) NUTS-3 area (Austria), and corresponding multi-annual aggregations visualizations and numerical values. See text for more details.

**Fig. 4:**
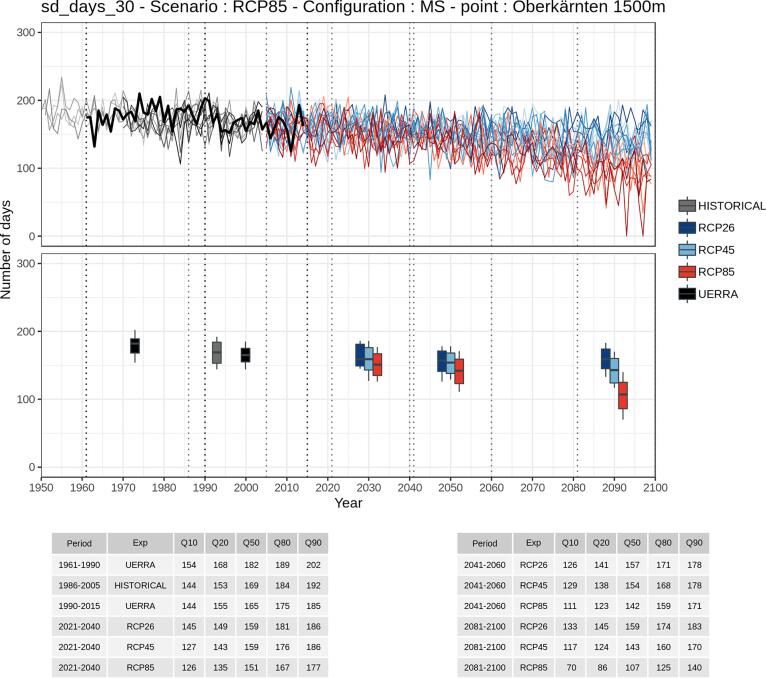
Same as 3 for managed snow (grooming .and snowmaking).

The primary approach to the annual-scale indicators consists in visualizing the time series of their evolution through time for a given NUTS-3 and elevation. Fig. 3, Fig. 4 illustrate the evolution in time of the number of days with more than 30 cm of snow on the ground for the Oberkärnten (Upper Carinthia) NUTS-3 area (Austria) at 1500 m elevation, for natural and managed (including snowmaking) conditions, respectively. The figures illustrate the time series from the reanalysis (thick black line), corresponding to the observed unfolding of the evolution of the conditions from 1960 to 2015, superimposed on climate projections for the historical time period (thin black lines) and future scenarios (thin colored lines). Each line corresponds to a GCM/RCM pair. This figure clearly illustrates the inter-annual variability in snow conditions, under past and future conditions, and how grooming and snowmaking reduce the variability for past and future conditions, and the magnitude of the decrease in indicator values for the 21st century, regardless of the climate scenario.

### 3.2 Geographical syntheses at given elevations, for various time periods and RCPs

**Fig. 5:**
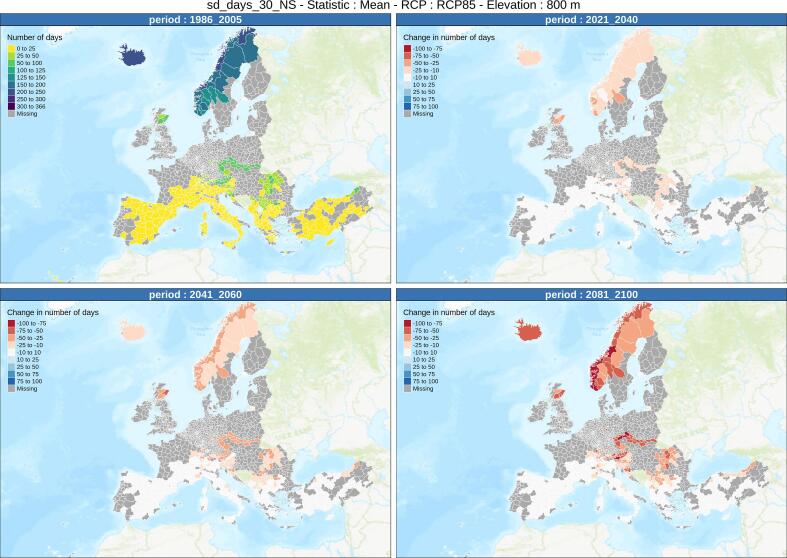
Evolution of the number of days with more than 30 cm of natural snow at 800 m elevation across Europe, based on climate projections, for the reference period 1986–2005 using historical simulations (top left corner), and relative change from the reference values for RCP8.5 projections for the time period 2021–2040 (top right), 2041–2060 (bottom left) and 2081–2100 (bottom right). The map displays values only for NUTS-3 areas where the selected elevation is included in the dataset. See text for more details.

**Fig. 6:**
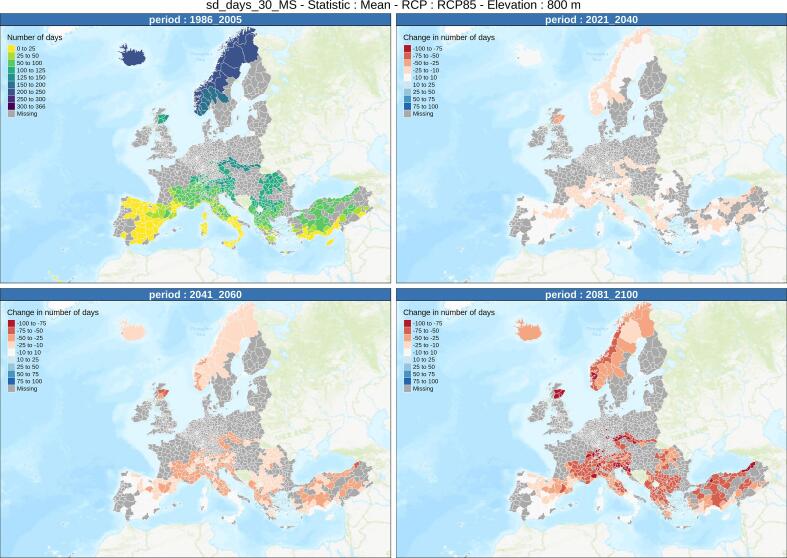
Same as 5 for managed snow (grooming and snowmaking).

In order to obtain a genuine pan-European perspective, and once a given elevation band is chosen, the series of indicators make it possible to compare future projections of the indicators, for various areas, and for given time periods into the 21st century, depending on the climate scenario. Fig. 5, Fig. 6 show the evolution of the number of days with more than 30 cm of natural and managed snow (grooming and snowmaking), respectively, at 800 m elevation across Europe, based on climate projections, between the reference period 1986–2005 using historical simulations, and future projections at various time periods in the 21st century using RCP8.5. A detailed analysis of these results is beyond the scope of this study, which instead focuses on the main features and outputs of the dataset.

### 3.3 Results as a function of elevation

**Fig. 7:**
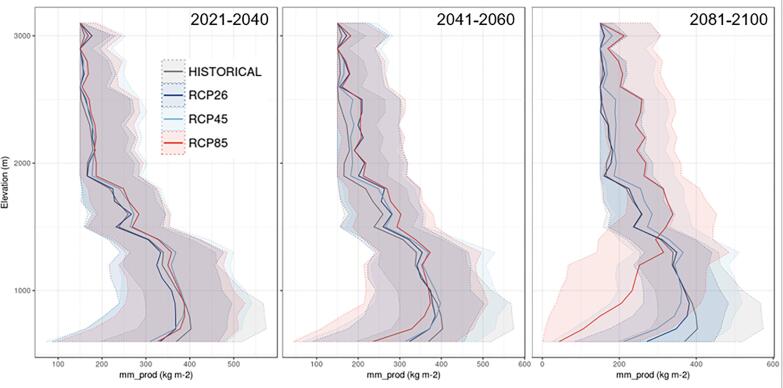
Simulated snow production for RCP2.6, RCP4.5 and RCP8.5 as a function of elevation for the NUTS-2 region Auvergne Rhône Alpes (France) for the time period 2021–2040 (left), 2041–2060 (middle) and 2081–2100 (right), compared to the historical time period (1986–2005, grey line on all plots). See text for more details.

As an example of a more elaborate use of the dataset, we illustrate the possibility to aggregate results from multiple NUTS-3 areas, e.g. for NUTS-2 areas, in order to discuss the elevation dependence of changes depending on the climate scenario. Fig. 7 illustrates the changes in simulated snow production for RCP2.6, RCP4.5 and RCP8.5 as a function of elevation for the NUTS-2 area Auvergne Rhône Alpes (France) for the time period 2021–2040, 2041–2060 and 2081–2100, compared to the historical time period (1986–2005). It clearly shows that at low elevation (below approximately 1300 m elevation) the amount of snow production decreases in climate projections, especially for end-of-century RCP8.5, because temperature conditions are increasingly unfavourable for snow production, and lower rates of changes above 1500 m elevation, with a trend to higher snow production at higher elevation for future climate change scenario, due to the fact that decreasing natural snow reliability, and sufficiently cold conditions during the main production phase (November and December), still allow for significant amounts of snowmaking. This trend is further strengthened for end-of-century RCP8.5 because snowmaking is then simulated to take place at increased pace to try to compensate for the decrease in natural snow conditions in this elevation range. These findings are in line with previous studies at the local and national scale (e.g., Spandre et al., 2019b, Scott et al., 2019, Steiger and Scott, 2020), although they are provided here homogeneously across all mountain areas of Europe.

### 3.4 Case studies

Several case studies were developed to foster the use of the pan-European products described above, for a range of stakeholders and geographical areas.

#### 3.4.1 Turkey

**Fig. 8:**
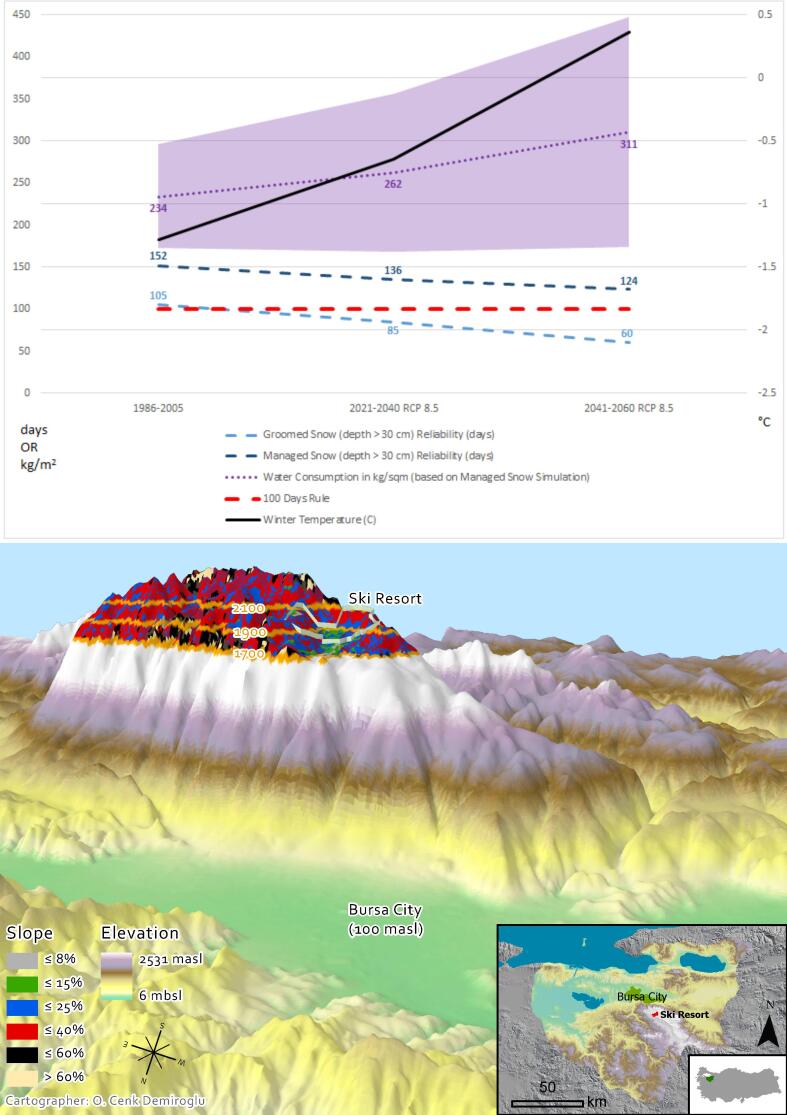
Illustration of the the Uludağ ski resort case study in Turkey. MTMSI results illustrating the winter temperature and snow reliability projections along with increased water consumption requirements for snowmaking at 1900–2000 m elevation band are shown on the upper graph. The black line represents the actual winter/ski season (NDJFMA) average temperature. The scene, created in ArcGIS Pro with a three times vertical exaggeration, shows the critical elevation bands for existing ski area as well as the slope diversity of existing and extendable ski areas.

This case study involved Austria-based ATC Mountain Tourism Consultants GmbH into exploring how the MTMSI data can support the master planning for the restructuring of the Uludağ ski resort in Western Turkey (Fig. 8) into a year-round mountain resort. Besides analyzing non-ski tourism development alternatives, ATC utilized the MTMSI dataset to assess the future snow reliability of the existing ski area, taking account of climate adaptation needs such as extension and/or snowmaking. The results (Fig. 8) show that the ski area is projected to lose its snow reliability in the near (2021–2040) and mid (2041–2060) ranges but could become technically reliable should snowmaking systems be installed. The two periods 2021–2040 and 2041–2060 indicated the business perspective that follows investment cycles and a regional development approach focusing on long term sustainability goals, respectively. Both periods follow the RCP8.5 trajectory to prepare for the worst. Data on annual amount of machine made snow produced was also retrieved to reflect on the water consumption resulting from the managed snow simulations and its financial and environmental implications. In this regard, the service projects a 311 ± 137 kg m^−2^ snow production to maintain a 124 ± 22 days ski season in the period 2041–2060, and such data was seen as a departure point for further calculations.

ATC was highly satisfied with what the MTMSI has to offer, as the company conventionally employs meteorological observations, and more recently, outsourced custom-made projections, when dealing with climatic suitability of mountain resorts and destinations. In Turkey, however, meteorological observations at high altitude locations are very rare. Moreover, basing investment and development decisions on observed climate conditions is considered misleading in a warming world. Custom-made projections, on the other hand, are costly and time-consuming, and usually based on a very limited number of GCM-RCM pairs. With the help of the service, ATC now had the timely and free access to readymade indicator results, based on multiple models and Representative Concentration Pathways, while some improvement needs were noted.

Use of the MTMSI was first tested by comparing its historical (1986–2005) natural snow cover projections against in situ observations available for 1877 m elevation of the ski resort (General Directorate of Meteorology, 2020). The multi-model mean of the projections at 1900–2000 m yielded a value of 103±29 days, slightly underestimating the 135 days average of the observations on its upper bound. While this was found highly satisfactory by ATC according to their needs, other critical elevation bands such as 1800–1900 m and 2000–2100 m turned out to have less realistic values, probably due to the fact that the representative reanalysis points were highly distant from the case site within the fairly extensive Bursa Province (NUTS-3 area). Moreover, no values existed for the critical extension bands of 2100–2500 m, as the highest points for Bursa Province are smoothed down to 2100 m under UERRA’s 5.5 km resolution. This was not much of a concern for this particular case, as northern faces of these higher elevation zones did not anyway offer much of the potential green and blue runs (Fig. 8), most demanded by the Turkish market, according to ATC. Last but not least, ATC enjoyed using the ”compare” tool of the service to account for vulnerability of Uludağ relative to its competitors in Turkey and the wider region, but identified a drawback from the lack of data for Turkey’s two provinces Van and Hakkari which promise climatically and topographically very high potentials for ski tourism development. A similar problem was also noted due to lack of coverage for the entire Caucasus. Such incompleteness resulted due to the extent limits of the EURO-CORDEX domain (Fig. 1).

#### 3.4.2 Switzerland

**Fig. 9:**
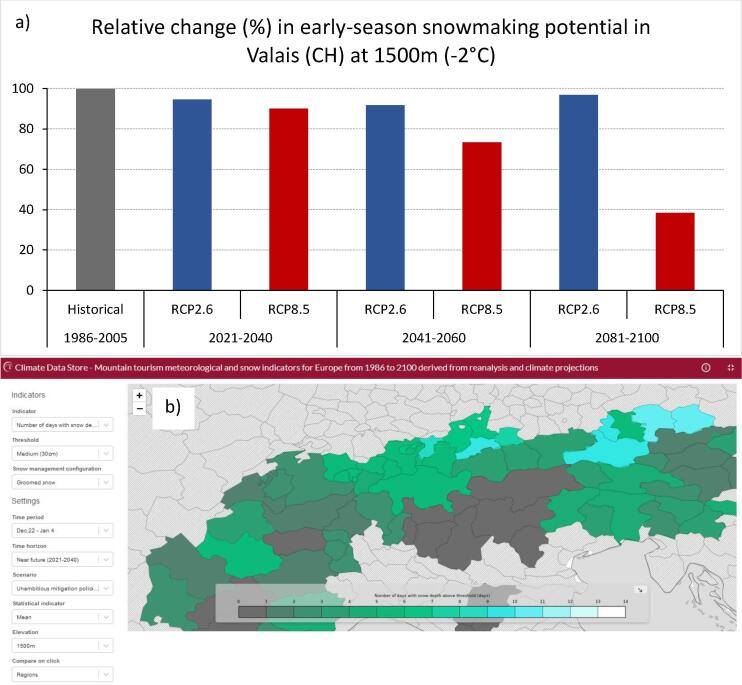
Illustration of the SBS case study in Switzerland. (a) Change in early-season potential snowmaking hours across time periods and RCPs. (b) Screenshot of the MTMSI app showing the number of days with more than 30 cm of groomed snow over the Christmas/New Year’s period (mean values; near future: 2021–2040; RCP8.5).

Seilbahnen Schweiz (SBS), the association of cable car companies in Switzerland, has 360 members (e.g. ski area operators) from all over the country. Major tasks include information/knowledge provision, communication and lobbying. Its target groups are its members (i.e. regional associations, individual businesses) but also policy makers, the media and the general public. The ski tourism industry has been repeatedly identified as being particularly vulnerable to climate change. State-of-the-art and customized climate information helps to cope with the impacts of climate change, and it is part of the Association’s business to make such information available to its members, in particular to the ski area operators. The case study is focusing on snow reliability and was set up to support the association in fulfilling its tasks, namely information/knowledge provision and communication. Two sets of indicator from the MTMSI application are chosen to demonstrate the benefits of the C3S European Tourism Service:

*Early-season potential snowmaking hours* It can be assumed that the ski areas will further invest in snowmaking capacity - be it to cover additional ski slopes or to increase the efficiency of existing facilities. This has to be done early in the season because the so called base-layer snowmaking takes place in November and December in order to guarantee a timely start of the season and to secure the economically critical Christmas-New Year’s period. Fig. 9a, based on MTMSI data, gives an example and shows the relative change (%) in early-season potential snowmaking hours (wbt-2-hrs indicator) in the Canton of Wallis/Valais (Switzerland) at 1500 m above sea level (NUTS-3: CH012) for the WBT lower than −2°C threshold, different time periods and two RCPs (2.6 and 8.5).

*Number of days with more than 30 cm of natural, groomed and managed snow* This is a standard indicator to investigate snow reliability. However, the MTMSI application features a series of valuable specifications and provides data on a pan-European level (based on a consistent methodology). This is very helpful as it allows for a convenient impact assessment (e.g. geographic comparisons), and the respective information can be easily communicated to the target groups in order to raise awareness, guide adaptation etc. Fig. 9b (screenshot), for example, shows the number of days with more than 30 cm of groomed snow over the Christmas/New Year’s period (mean values; near future: 2021–2040; RCP8.5). This corresponds to the indicator sd-days-Xmas-GS.

#### 3.4.3 Andorra

The case study was developed in cooperation with the National Energy and Climate Change Office of the Andorra Government. The case explored how the MTMSI data could help to the National Climate Change Adaptation Strategy specially to assess and design the necessity of sectoral adaptation strategies. The relevance of the indicators was explored and introduced in the process to design the national and sectoral adaptation strategy of the national report to UNFCCC within a few years. The MTMSI indicators permitted to directly explore the future evolution under different climate change scenarios of automatically computed relevant indicators for the ski industry. In this specific case study indicators relevant for future ski operations such as days of managed snow depth above the 30 cm threshold or starting and ending dates were visualized to assess future changes and their impact on future operations in ski resorts. The case study enabled a general assessment contributing to the adaptation strategy of the Andorran ski industry, compared to other European ski areas, and some insight about the local vulnerability for ski operations and snowmaking in Andorra.

The MTMSI App was used to identify generally the operation conditions (snow days above operational threshold and starting and end season date) in future climate change scenarios to assess the vulnerability of Andorra as a ski destination and infer with the available data the potential future impact to the 2 ski resorts. To infer the local conditions to ski resorts, elevation ranges were used from the data to identify the changes in these specific conditions for the selected indicators.

As the main current adaptation strategy for the Andorran ski resorts, the future change in the snowmaking conditions was analyzed thanks to the produced indicators about snowmaking hours in early season and total yearly snowmaking production.

Data of snow depth (days with natural snow, groomed and managed > 30 cm threshold and days with Snow water equivalent > 100 kg m^2^ of managed snow; start and end season date) was analyzed for the reference/past period and for the RCP4.5 and RCP8.5 for mid and end-century. Data of snowmaking production capacity (total yearly snowmaking production and early-season snowmaking hours) was analyzed for the reference/past period and for the RCP4.5 and RCP8.5 for mid and end-century.

Then, maps of the snow depth indicators were generated to visualize the temporal evolution at different RCP scenarios at country scale for mean elevation conditions. Maps of the snowmaking production indicators were generated to visualize the temporal evolution at different RCP scenarios at country scale for mean elevation conditions.

**Fig. 10:**
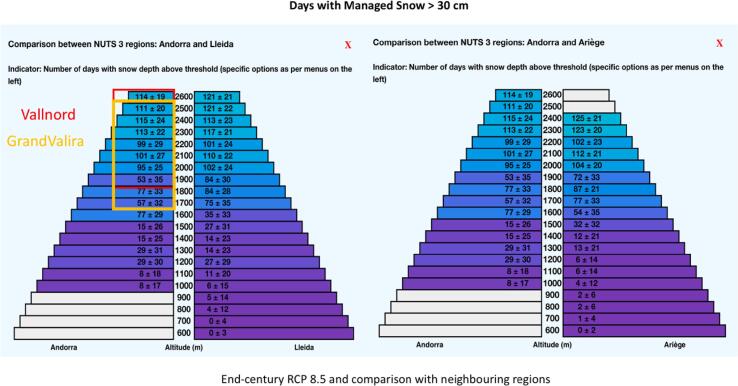
Comparison of the number of days with more than 30 cm of managed snow (sd-days-30-MS indicator) between Andorra and Lleida, Spain (left) and Ariège, France (right) for end of century RCP8.5. The figure highlights the elevation range for the GrandValira and Vallnord ski resorts.

Finally values for the indicators were inferred for the local conditions from the data sets and visualizations. To infer these values at the potential local conditions of the 2 Andorran ski resorts, the different indicators and their evolution at mid and end-century for RCP4.5 and RCP8.5 were visualized and analyzed at elevation range for the Andorra/NUTS-3 polygon. Fig. 10 shows the comparison between Andorra and neighboring NUTS-3 areas of Ariège (France) and Lleida (Spain) of days with more than 30 cm of managed snow (sd-days-30-MS indicator) in different elevation bands for the RCP 8.5 scenario at the end of the century and showing the elevation range of GrandValira and Vallnord ski resorts.

## 4 Discussion

This contribution describes the workflow conducive to the production of a pan-European dataset of climate change impact indicators for ski tourism. This comprehensive dataset was designed through a co-design process involving ski tourism stakeholders. This Copernicus Climate Change Service - Sectoral Information System for European Tourism has been made available through the corresponding catalogue entries on the Climate Data Store (CDS) since the Summer 2020.

**Fig. 11:**
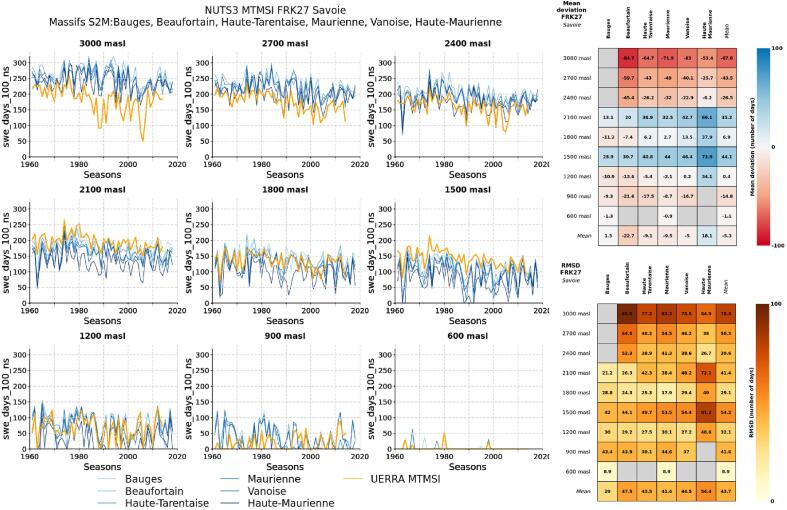
Comparison of the number of days with more than 100 kg m^−2^ SWE for natural snow between the MTMSI dataset, for the Savoie NUTS-3 (Northern French Alps) and the S2M reanalysis for the ”massifs” included in this NUTS-3 area. Time series for the time period 1960–2019 (left hand side) and evaluation metrics for each elevation band (mean deviation and root mean square deviation, right hand side).

The main principles and chain of models used for this product were used in previous studies, using similar climate projection input data (Verfaillie et al., 2018, Spandre et al., 2019a, Spandre et al., 2019b) and are considered state-of-the-art. The UERRA 5.5 km reanalysis has undergone evaluation as part of the UERRA European Project (Bazile et al., 2017), and an assessment of its temperature dataset in the Swiss mountain areas showed no major deficiency although high elevation values can significantly deviate from some observations (Scherrer, 2020). Here we consider that, due to the larger amount of input data, the use of a true surface reanalysis system (including precipitation analysis), and the higher resolution (5.5 km) than previous products (e.g, E-OBS), this dataset is more fit-for-purpose than alternative products at the European scale. Nevertheless, the quantity and quality of input data to the UERRA 5.5 km varies across the domain, so that heterogeneities in the dataset are likely (see https://confluence.ecmwf.int/display/UER/Issues+with+data). However, this dataset probably constitutes the best available data set at European scale with capabilities to drive impact models such as energy-balance snow cover models, but still subject to significant improvements for the future (Bazile et al., 2017, Scherrer, 2020). Due to the absence of a sufficiently long and geographically broad reference observation dataset, which could be used to evaluate the MTMSI product, a formal evaluation of the MTMSI dataset has not been carried out in detail. Fig. 11 provides a comparison between the MTMSI dataset for the time period 1960–2015 for the NUTS-3 region Savoie, France, and the SAFRAN-Crocus (S2M) reanalysis (Durand et al., 2009, Vernay et al., 2019), which has a smaller space resolution than the MTMSI data and uses more in situ observations than the UERRA reanalysis. The S2M reanalysis provides data for ”massifs” and by steps of 300 m elevation, therefore each NUTS-3 area in the French Alps and Pyrenees can include several massifs, and data can be compared directly only every 300 m elevation. Here we focus on the number of days with more than 100 kg m^−2^ natural snow SWE. The Savoie NUTS-3 area is located in the Northern French Alps, and hosts most large ski resorts in France (approximately 40 % of ski tourism infrastructures in France). Additional such figures for other NUTS-3 areas in France are provided in Annex. The comparison shows that in many cases, the indicator values for MTMSI and S2M follow similar patterns, although systematic deviations can be found, depending on the NUTS-3 area and the elevation. In most cases, main deviations are found at high elevation, where MTMSI values are generally lower than S2M (i.e., less snow in MTMSI than in S2M). These deviations are consistent with the lower resolution of the UERRA dataset (5.5 km), and the low density of precipitation observation networks at high elevation – often associated with underestimation of solid precipitation. In coming years, emerging datasets could be used to further evaluate the MTMSI dataset against in situ or satellite observations at a wider geographical scale (e.g., Gascoin et al., 2019, Matiu et al., 2021).

Uncertainties from climate projections are accounted for using a multi-model approach, which uses a larger number of GCM/RCM pairs than previous studies at the pan-European scale (Table 1), and comparable number to local-scale studies (e.g., Marke et al., 2014, Spandre et al., 2019a, Spandre et al., 2019b). The snow cover model Crocus has demonstrated state-of-the-art performances in model intercomparison exercices (Krinner et al., 2018). Within the scope of this product, the main limitation to the use of the Crocus model is the absence of several processes shaping the snow cover on ski slopes (e.g. wind-drifting, skiers erosion), and the use of a single set of snow management parameters (grooming, snowmaking) across Europe (Abegg et al., 2020, Hanzer et al., 2020), although regional variations in management practices, such as snowmaking practices (e.g. temperature thresholds, production targets, starting and ending dates for snowmaking) already occur and are likely to evolve in the 21st century, due to changes in technology and adaptation of management practices to climate change.

**Fig. 12:**
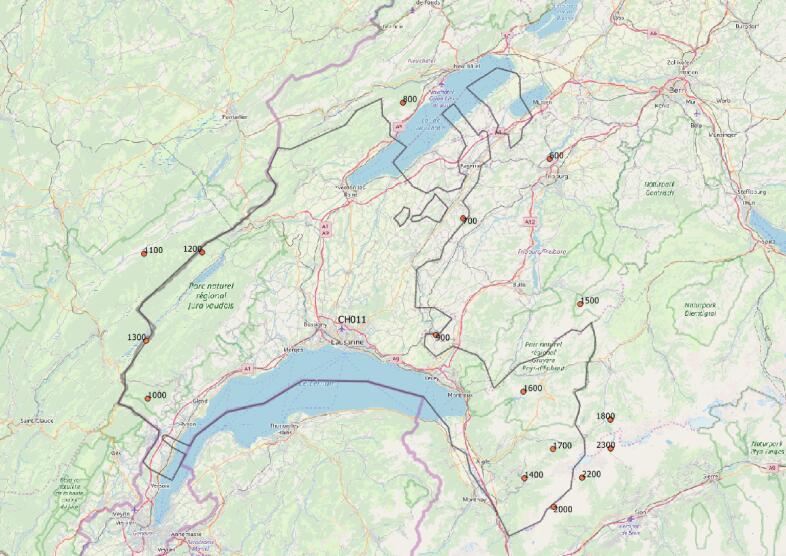
Illustration of the representative points within the UERRA5.5 km reanalysis attributed to each elevation band, in the example of the Vaud canton in Switzerland (NUTS-3 CH011).

The main issue/caveat identified on this data set is related to the geographical setting employed. Indeed, there is a trade-off between the representation of spatial variability within mountain ranges and the specification of a pan-European product with features as homogeneous as possible. In order to operate on a manageable number of points, while representing the elevation dependence of changes in the mountain environment, the choice was made to define the indicators on NUTS-3 areas. This is consistent with previous studies, and makes it possible to combine the indicators with other socio-economic indicators. However, given the size of NUTS-3 areas, it was necessary to select points, in order to represent 100 m elevation bands, which are sometimes over 100 km apart. NUTS-3 are purely administrative borders, which implies that they sometimes do not align with the physical geography and local climate zoning. Fig. 12 shows an example of the selection of UERRA 5.5 km points for the Vaud canton in Switzerland (NUTS-3 CH011). It shows that sometimes UERRA-5.5 km points used for different elevation bands can be located quite far away. While for each point the climate projection information remains adequate, in such cases, the vertical lapse rate of the indicators can be nonlinear, because, while elevation is the primary control for mountain climate, smaller scale processes operate within NUTS-3 areas. Alternatives for this caveat were sought, but not achieved within the time frame of the production schedule. For example, it was not possible to combine several points within a given NUTS-3 for a given elevation and perform some spatial averaging: this would have smoothed out meteorological conditions, in particular precipitation events, and resulted in unrealistic snow model outputs both for reanalysis and climate projections. As a consequence, care should be exercised when analyzing small scale features such as the elevation dependence of the indicators within a given NUTS-3 or differences between neighboring NUTS-3 areas for a given elevation. We also note that the EURO-CORDEX geographical domain does not cover the Easternmost mountain provinces of Turkey.

Despite these shortcomings and limitations, the MTMSI dataset provides information relevant to the impact of climate change on ski resorts operating conditions in Europe far beyond state-of-the-art existing products at this geographical scale. Insights from case studies lead to the following recommendations for users:

• remain aware of the key features and limitations of the MTMSI dataset, in particular the fact that it provides data on horizontal terrain, which needs to be taken into account when assessing past and future snow cover changes on ski slopes;
• combine information from MTMSI with local snow cover information, in order to assess potential deviations with past observations and link horizontal and sloping terrain;
• focus on changes in indicator values over time (from past to future climate conditions) rather than on absolute values, which alleviates some of the drawbacks of the limitations of the MTMSI dataset.

## 5 Conclusions

The C3S European Tourism MTMSI provides a pan-European, homogeneously produced set of indicators relevant to the impact of climate change on ski tourism operating conditions in Europe. It makes it possible to quantify the impacts, as a function of elevation, arising from various climate change scenarios (RCP2.6, RCP4.5 and RCP8.5) for several time periods in the future (2021–2040, 2041–2060 and 2081–2100), and taking into account snow management practices such as grooming and snowmaking. While this data set fills a knowledge gap at the regional scale, it is not meant to replace higher resolution products which are available in some European countries or local areas, and provide a more detailed view of future snow conditions in some ski resorts, accounting, for example, for slope, aspect, local phenomena and local snow management practices. However, given that the workflow for the generation of the product is homogeneous at the pan-European level, this product is useful to compare the main features of past and future snow conditions at the pan-European level, or to compare distant destinations (e.g., compare Scandinavia and Eastern Europe for a given elevation and time horizon). Furthermore, where no other source of information is available, it provides an original outlook on future meteorological and snow conditions in mountain areas.

It is the first version of a pan-European product for mountain (ski) tourism under climate change, which holds significant potential for applications but can be improved in several areas, such as:

• Refined methodology for location/elevation issues,
• Improvements of the regional reanalysis and use of more GCM/RCM pairs,
• Improvements of the adjustment method,
• Further improvements of the snow cover model.

We also note that, while the product was developed within the C3S European Tourism Sectoral Information System, and is referred to as Mountain Tourism, ski tourism only covers a fraction, yet a significant and highly climate-sensitive, of the full breadth of tourism in mountain areas. Based on the dataset generated for this set of indicators, additional indicators could be developed for year-round mountain tourism, or for complementary winter tourism indicators. The MTMSI dataset is also relevant to other sectors and domains in the mountain environment beyond the tourism sector, pending dedicated evaluation of its strengths and weaknesses for other applications.

## Data and code availability

The MTMSI dataset is available on the Copernicus Data Store following this doi: https://doi.org/10.24381/cds.1ac1b4ba under the Copernicus licence. The Crocus snow cover model used for this work is developed inside the open-source SURFEX project (http://www.umr-cnrm.fr/surfex/, last access: 7 June 2020). For reproducibility of results, the version used in this work is tagged as ”C3S-European-Tourism-MTMSI-2019” on the SURFEX git repository.

## CRediT authorship contribution statement

**Samuel Morin:** Conceptualization, Funding acquisition, Investigation, Methodology, Software, Supervision, Validation, Writing - original draft, Writing - review & editing. **Raphaëlle Samaco**ï**ts:** Data curation, Investigation, Software, Visualization, Writing - original draft. **Hugues François:** Data curation, Conceptualization, Investigation, Methodology, Software, Validation, Visualization, Writing - original draft, Writing - review & editing. **Carlo M. Carmagnola:** Investigation, Methodology, Software, Writing - original draft, Writing - review & editing. **Bruno Abegg:** Conceptualization, Investigation, Methodology, Supervision, Writing - original draft, Writing - review & editing. **O. Cenk Demiroglu:** Investigation, Methodology, Supervision, Visualization, Writing - original draft, Writing - review & editing. **Marc Pons:** Investigation, Methodology, Supervision, Writing - original draft, Writing - review & editing. **Jean-Michel Soubeyroux:** Conceptualization, Investigation, Project administration, Supervision. **Matthieu Lafaysse:** Methodology, Methodology, Writing - original draft. **Sam Franklin:** Visualization. **Guy Griffiths:** Visualization. **Debbie Kite:** Conceptualization, Methodology, Visualization, Writing - original draft. **Anna Amacher Hoppler:** Conceptualization, Investigation, Methodology, Supervision, Writing - original draft, Writing - review & editing. **Emmanuelle George:** Conceptualization. **Carlo Buontempo:** Conceptualization, Project administration, Writing - original draft. **Samuel Almond:** Conceptualization, Data curation, Project administration, Validation. **Ghislain Dubois:** Conceptualization, Data curation, Project administration, Supervision. **Adeline Cauchy:** Conceptualization, Data curation, Project administration, Supervision, Writing - original draft.

## Declaration of Competing Interest

The authors declare that they have no known competing financial interests or personal relationships that could have appeared to influence the work reported in this paper.
